# Dual genetic mechanisms of heterosis: population structure and gene action

**DOI:** 10.3389/fpls.2025.1715826

**Published:** 2026-01-27

**Authors:** Fernando S. Aguilar, Kendall R. Lamkey, Jode W. Edwards

**Affiliations:** 1 Department of Agronomy, Iowa State University, Ames, IA, United States; 2 College of Agriculture and Life Sciences, Iowa State University, Ames, IA, United States; 3 Corn Insects and Crop Genetics Research Unit, United States Department of Agriculture, Agricultural Research Service, Ames, IA, United States

**Keywords:** breeding, dominance, heterosis, maize, quantitative genetics

## Abstract

**Introduction:**

Heterosis refers to the superiority of a hybrid over its parents. Existing heterosis theory has not sufficiently addressed the contribution of inbreeding at both population level and the level of individual lines within populations. The objectives of the present paper were to formalize theoretical extensions of heterosis theory to address inbreeding at multiple levels, to empirically test the theory in maize, and to provide greater clarity in the quantitative genetic interpretation of heterosis as a function of independent genetic principles of population structure and gene action.

**Methods:**

Existing heterosis theory for biparental crosses was extended by adding terms for inbreeding within panmictic parent populations. The theory was tested with an experiment in maize with a diverse set of panmictic and inbred parents.

**Results:**

Extended theory demonstrated that both heterosis and inbreeding depression are linear functions of inbreeding, *F_ST_* at the population level, and *f* at the individual level, under a model of directional dominance. The model demonstrates that heterosis is expected to be negatively related to both midparent value and inbreeding depression within parent populations, i.e., heterosis increases as midparent value decreases and as inbreeding depression within parent populations decreases. Consistent with theoretical predictions we found that that for maize grain yield midparent value predicted 86% of heterosis in a set of crosses and parental inbreeding depression predicted 70% of variation in heterosis among crosses.

**Discussion:**

Model extensions presented here illustrate the excess and transient nature of heterozygosity in the F_1_ generation that is partially responsible for the unique performance benefit of F_1_ hybrids. Mechanistically, the theory illustrates that heterosis is a function of two separate and independent mechanisms, population structure and gene action, both of which need to be considered in understanding the mechanisms of heterosis.

## Introduction

George Shull (1914) first adopted the term heterosis in reference to the “stimulating effect of hybridity.” In modern usage, heterosis is defined more specifically to refer to a contrast between parental and hybrid generations reflected by an increase in the mean trait values of hybrid individuals for a wide range of phenotypes but those related to stature and growth in particular (Charlesworth and Willis, 2009; Falconer and Mackay, 1996; Lamkey and Edwards, 1999; Shull, 1914; Willham and Pollak, 1985). In quantitative genetic models, heterosis is a function of nonadditive gene action and a difference in heterozygosity between cross and parental generations (Falconer and Mackay, 1996; Hill, 1982; Lamkey and Edwards, 1999; Lynch, 1991; Willham and Pollak, 1985). The primary emphasis of most heterosis research, especially in plants, has been on the contribution of gene action, with much of the early emphasis on the role of dominance and the ensuing debate between dominance and overdominance (Crow, 1952; Crow, 1948, Crow, 1998; Crow, 2000; Jones, 1917; Keeble and Pellew, 1910; Sprague, 1983). Recently, the emphasis has shifted to the search for the molecular basis of heterosis (Baldauf and Hochholdinger, 2023; Birchler et al., 2003; Blum, 2013; Goff, 2011; Das et al., 2021; Kaeppler, 2011, Kaeppler, 2012; Lippman and Zamir, 2007; Mackay et al., 2021; Rehman et al., 2021; Springer and Stupar, 2007; Swanson-Wagner et al., 2006; Wu et al., 2021). While research on gene action has heavily focused on dominance, epistasis can also make important contributions to heterosis, especially in advanced generations of crossing (Dickerson, 1972; Hill, 1982; Lynch, 1991; Willham and Pollak, 1985). Epistasis may result in outbreeding depression in crosses between highly divergent populations and/or between species (Moll et al., 1965; Lynch, 1991). Epistasis has received much less attention in heterosis research than dominance, perhaps because of the complexity of measuring epistasis and the fact that epistasis generally accounts for much less genetic variation than dominance.

The role of population structure in heterosis has received far less attention than gene action, to the point where it is mostly ignored in past research, in which the focus is solely on gene action and molecular mechanisms. However, heterosis does not occur in the absence of a difference in heterozygosity between the parents and crosses created by genetic divergence. Different parameterizations have been used to quantify the difference in heterozygosity between parents and crosses in the existing theory, including a hybridity index (Lynch, 1991), divergence in allele frequency (Falconer and Mackay, 1996; Lamkey and Edwards, 1999; Willham, 1970; Willham and Pollak, 1985), and Mendelian segregation ratios (Hill, 1982). In all parameterizations, the contribution of intralocus dominance to heterosis was a linear function of the dominance coefficient and increased heterozygosity between parents and cross-generations. In the Willham and Pollak (1985) parameterization, for example, the change in heterozygosity is represented by Δ^2^ the squared difference in allele frequency between parent populations. Divergence in allele frequency between parent populations can be driven by genetic drift, selection, or mutation; however, in most systems, drift is likely the most important driving force. Under a model of genetic drift, divergence in allele frequency is a linear function of increased inbreeding within parent populations, quantified by Wright’s *F_ST_* (Wright, 1951), which is the probability of identity by descent within subpopulations relative to a base population. Increased inbreeding within populations also leads to inbreeding depression, which has been referred to as the opposite or complement of heterosis (Falconer and Mackay, 1996; Labroo et al., 2021; Mackay et al., 2021), highlighting the fact that both are linear functions of heterozygosity.

Prior analyses of heterosis have dealt with genetic divergence resulting from a single level of inbreeding at the population level. In applied breeding programs, inbreeding can occur at several levels. In species with well-established inbred-hybrid breeding systems, such as maize, breeding germplasm is organized into subpopulations of related lines referred to as heterotic groups (Tracy and Chandler, 2006). Inbreeding occurs at two levels in these systems, with a heterotic group being an inbred subpopulation, characterized by *F_ST_ >*0, with inbred lines being extracted from heterotic groups with *F* = 1. Hybrids are crosses between inbred lines from different heterotic groups. Most existing heterosis theories have modeled divergence as a function of inbreeding at the population level but have not included additional levels of inbreeding. Lamkey and Edwards (1999) extended the theory described by Willham and Pollak (1985) to include inbreeding within parent populations to represent the derivation of inbred lines within populations such as heterotic groups. Lamkey and Edwards (1999) partitioned heterosis into two sources due to two levels of inbreeding. In most previous studies, with one level of inbreeding, a linear association between heterosis and heterozygosity was apparent. However, in the partitioning of heterosis by Lamkey and Edwards (1999), additional terms were introduced to model the additional level of inbreeding that somewhat obscured the linear relationship between heterozygosity and heterosis across the two levels of inbreeding.

The objectives of the present study were to formalize the theoretical extensions presented by Lamkey and Edwards (1999) to delineate the relationships among heterosis, inbreeding depression, and heterozygosity more clearly, to empirically test the theory in maize, and to provide greater clarity in the quantitative genetic interpretation of heterosis as a function of independent genetic principles of population structure and gene action.

## Materials and methods

### Genetic model

**Figure 1:**
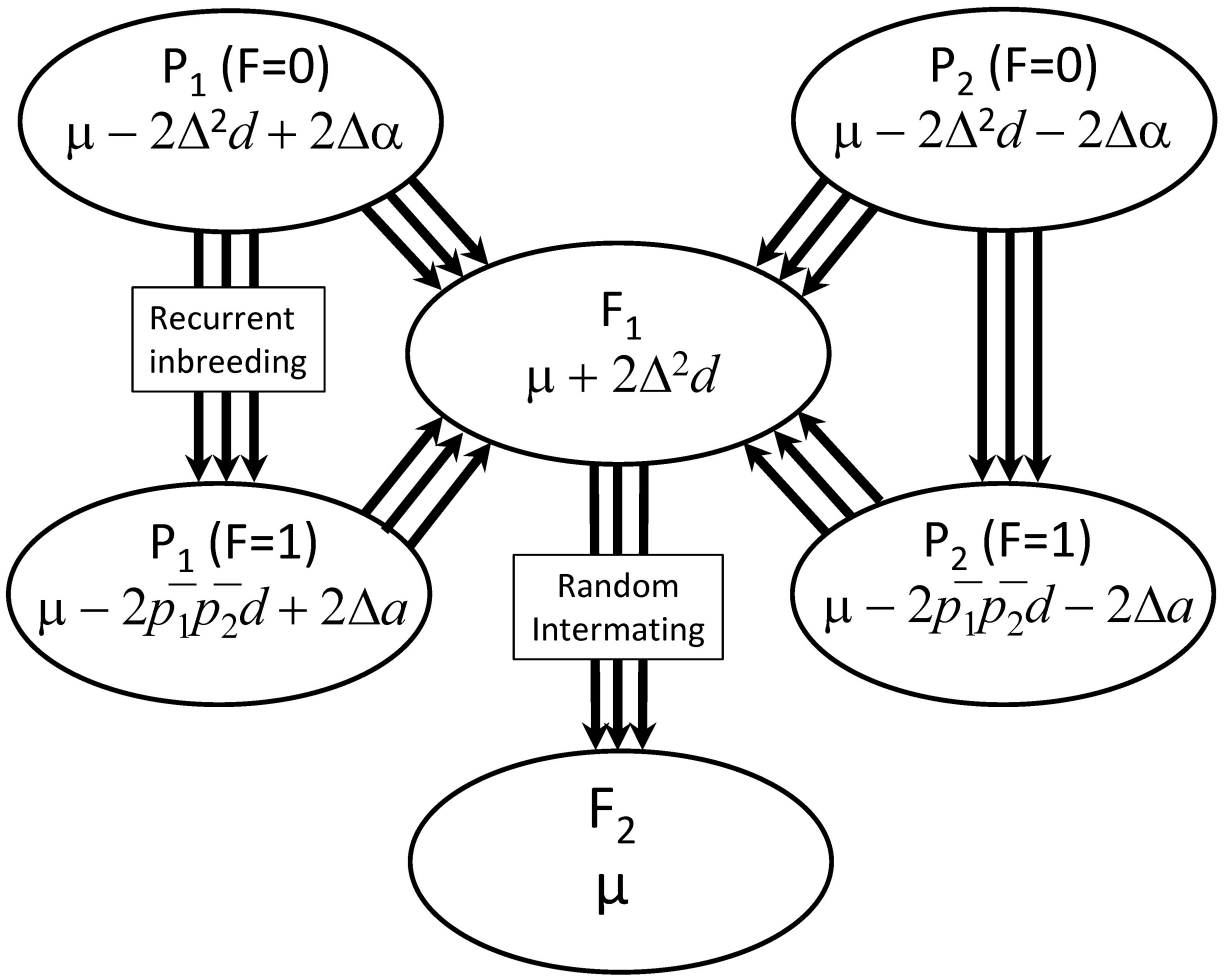
Mating scheme showing parental, cross, and inbred generations with their expected values. Arrows between populations represent the crossing or inbreeding of multiple individuals to generate crossbred or inbred generations.

Willham and Pollak (1985) developed theory for midparent heterosis in a cross between two subpopulations differing in allele frequencies (**Figure 1**). The reference population in which allelic frequencies and effects were defined was the F_2_ generation obtained by one generation of intermating of the F_1_-hybrid cross between parental subpopulations (Willham and Pollak, 1985; **Figure 1**). Allelic frequencies of allele A*_i_* in the parental subpopulations were denoted by *p_i_* for parent 1 and *p_i_*’ for parent 2. These frequencies were reparameterized using the average allele frequency of the parents (*p̅*_i_ = ½(*p_i_* + *p_i_*’)) and half the difference in frequency between parents, *δ_i_*, (*δ_i_* = ½(*p_i_* − *p_i_*’)), such that frequency of allele A_*i*_ was *p̅_i_* in the F*_2_* reference population, *p_i_* = *p̅_i_* + *δ_i_* in parent 1 (P_1_) and *p_i_*’ = *p̅_i_* − *δ_i_* in parent 2 (P_2_). Multiplying allelic arrays for parents by genotypic value of genotype A*_i_*A*_j_* resulted in the following expected values for six parental and cross generations (Willham and Pollak, 1985):

(1) $$\begin{array}{l} {\text{F}}_{2}=\sum_{ij}{\bar{p}}_{i}{\bar{p}}_{j}Y_{ij}\\ {\text{P}}_{1}=\sum_{ij}({\bar{p}}_{i}+\delta_{i})({\bar{p}}_{j}+\delta_{j})Y_{ij}=\sum_{ij}{\bar{p}}_{i}{\bar{p}}_{j}Y_{ij}+\sum_{ij}({\bar{p}}_{i}\delta_{j}+{\bar{p}}_{j}\delta_{i})Y_{ij}+\sum_{{\;}_{i}}\sum_{j}\delta_{i}\delta_{j}Y_{ij}\\ {\text{P}}_{2}=\sum_{ij}({\bar{p}}_{i}-\delta_{i})({\bar{p}}_{j}-\delta_{j})Y_{ij}=\sum_{ij}{\bar{p}}_{i}{\bar{p}}_{j}Y_{ij}-\sum_{ij}({\bar{p}}_{i}\delta_{j}+{\bar{p}}_{j}\delta_{i})Y_{ij}+\sum_{{\;}_{i}}\sum_{j}\delta_{i}\delta_{j}Y_{ij}\\ {\text{F}}_{1\;(\text{P}1\times \text{P}2)}=\sum_{ij}({\bar{p}}_{i}+\delta_{i})({\bar{p}}_{j}-\delta_{j})Y_{ij}=\sum_{ij}{\bar{p}}_{i}{\bar{p}}_{j}Y_{ij}-\sum_{i}\sum_{j}\delta_{i}\delta_{j}Y_{ij}\\ \text{B}{\text{C}}_{\text{PI}}=\sum_{ij}({\bar{p}}_{i}+\delta_{i}){\bar{p}}_{j}Y_{ij}=\sum_{ij}{\bar{p}}_{i}{\bar{p}}_{j}Y_{ij}+\sum_{ij}{\bar{p}}_{j}\delta_{i}Y_{ij}\\ \text{B}{\text{C}}_{\text{P}2}=\sum_{ij}{\bar{p}}_{i}({\bar{p}}_{j}-\delta_{j})Y_{ij}=\sum_{ij}{\bar{p}}_{i}{\bar{p}}_{j}Y_{ij}-\sum_{ij}{\bar{p}}_{i}\delta_{j}Y_{ij} \end{array}$$

These expressions were simplified by collecting like terms and assuming a two-allele model. For purposes of modeling generation means, a two-allele model is much more interpretable and results in an identical set of parameters with the same coefficients as a multi-allele model. Genotypic values were *Y*_11_=*a*, *Y*_12_=*d*, and *Y*_22_=–*a* such that *a* is one half the difference in genotypic value between homozygotes and *d* is the heterozygote deviation from midpoint between homozygotes (assumed zero). The six expressions (1) given for the six generations were functions of just three terms ∑*_ij_p̅_i_p̅_j_Y_ij_*, ∑*_ij_p̅_i_δ_j_Y_ij_*, and ∑*_i_*∑*_j_δ_i_δ_j_Y_ij_* (middle term is symmetrical and identical to ∑*_ij_δ_i_p̅_j_Y_ij_*). The first term, ∑*_ij_p̅_i_p̅_j_Y_ij_*, is contained in every expression and is equivalent the F_2_ generation mean, denoted by μ = *a*(*p̅*_1_ - *p̅*_2_) + 2*p̅*_1_*p̅*_2_*d*. The second and third terms were reduced in a 2-allele case by defining Δ as half the difference in allele frequencies between the two parents with only 2 alleles, Δ = [½(*p_i_* – *p_i_*’)], which reduced *δ*_1_ and *δ*_2_ to Δ and -Δ, respectively (Willham and Pollak, 1985). Substituting Δ for δ*_i_* and δ*_j_* and numerical subscripts for genotypic values in the two-allele case resulted in ∑*_ij_p̅_i_δ_j_Y_ij_* = Δ[*p̅*_1_(*Y*_11_ – *Y*_21_) + p̅_2_(*Y*_12_ – *Y*_22_)] = Δ*α*, where *α* is the average effect of an allelic substitution, and ∑*_ij_δ_i_δ_j_Y_ij_* = Δ^2^(*Y*_11_ - *Y*_21_ – *Y*_12_ + *Y*_22_) = 2Δ^2^*d* (Willham and Pollak, 1985). Substitutions produced the following simplification of expressions in Equation 1:

(2) $$\begin{array}{l} {\text{F}}_{2}=\;\mu \\ \begin{array}{l} {\text{P}}_{1}=\mu +2\text{Δ}\alpha -2{\text{Δ}}^{2}d\\ {\text{P}}_{2}=\mu -2\text{Δ}\alpha -2{\text{Δ}}^{2}d\\ {\text{F}}_{1}=\mu +2{\text{Δ}}^{2}d\\ \text{B}{\text{C}}_{\text{P}1}=\mu +\text{Δ}\alpha \\ \text{B}{\text{C}}_{\text{P}2}=\mu -\text{Δ}\alpha \end{array} \end{array}$$

Willham and Pollak (1985) developed their theory in the context of animal breeding where individual inbreeding within populations is not common. Lamkey and Edwards (1999) considered practices in plant breeding in which the development of inbred lines is common and extended Willham and Pollak’s theory to include any level of inbreeding (*f*) in any of the 6 generations. In the Lamkey and Edwards (1999) model, means of generations were expressed as weighted averages of panmictic population means (given in Equation 1) and inbred population means with weights of 1-*f* on the panmictic population mean and *f* on the inbred population mean. An inbred population mean was defined as the mean of a large sample of inbred individuals derived from a population (**Figure 1**). Inbred populations were composed entirely of homozygotes with frequencies of homozygous genotypes equivalent to allele frequencies in the noninbred population. For example, the mean of parent 1 was P_1_ = ∑*_i_*(*p̅_i_* +*δ_i_*)*Y_ii_* = (*p̅*_1_ + Δ)*a* + (*p̅*_2_ − Δ)(−*a*) = *a*(*p̅*_1_ − p̅_2_) + 2Δ*a* = *μ* − 2*p̅*_1_*p̅*_2_*d* + 2Δ*a.* The remainder of inbred generation means were derived by similar means:

(3) $$\begin{array}{l} {\text{F}}_{2}={\text{F}}_{1}={\bar{p}}_{1}a-{\bar{\text{p}}}_{2}a=\mu -2{\bar{p}}_{1}{\bar{p}}_{2}d\\ {\text{P}}_{1}=\mu -2{\bar{p}}_{1}{\bar{p}}_{2}d+2\text{Δ}a\\ {\text{P}}_{2}=\mu -2{\bar{p}}_{1}{\bar{p}}_{2}d-2\text{Δ}a\\ \text{B}{\text{C}}_{\text{P}1}=\mu -2{\bar{p}}_{1}{\bar{p}}_{2}d+\text{Δ}a\\ \text{B}{\text{C}}_{\text{P}2}=\mu -2{\bar{p}}_{1}{\bar{p}}_{2}d-\text{Δ}a \end{array}$$

The difference between panmictic F_2_ mean and inbred-F_2_ mean, 2*p̅*_1_*p̅*_2_*d*, is equivalent to inbreeding depression in the F_2_ reference population. Means of populations with general levels of inbreeding, *f*, (0 <*f* < 1) were obtained by taking a weighted average of panmictic means in Equation 2 and in red means in Equation 3:

(4) $$\begin{array}{l} {\text{F}}_{2}=\mu -2f{\bar{p}}_{1}{\bar{p}}_{2}d\\ {\text{P}}_{1}=\mu -2f{\bar{p}}_{1}{\bar{p}}_{2}d-2(1-f){\text{Δ}}^{2}d+2(1-f)\text{Δ}\alpha +2f\text{Δ}a\\ {\text{P}}_{2}=\mu -2f{\bar{p}}_{1}{\bar{p}}_{2}d-2(1-f){\text{Δ}}^{2}d-2(1-f)\text{Δ}\alpha -2f\text{Δ}a\\ {\text{F}}_{1}=\mu -2f{\bar{p}}_{1}{\bar{p}}_{2}d+2(1-f){\text{Δ}}^{2}d\\ \text{B}{\text{C}}_{\text{P}1}=\mu -2f{\bar{p}}_{1}{\bar{p}}_{2}d+(1-f)\text{Δ}\alpha +f\text{Δ}a\\ \text{B}{\text{C}}_{\text{P}1}=\mu -2f{\bar{p}}_{1}{\bar{p}}_{2}d-(1-f)\text{Δ}\alpha -f\text{Δ}a \end{array}$$

This theory was extended to include inbred lines as parents. In contrast to inbred populations, which are heterogeneous groups of inbred individuals, inbred lines are homogeneous groups of inbred individuals in which all individuals are genetically identical. The allele frequency of an inbred line is either zero or one, resulting in values for the average allele frequency and the difference in allele frequency of one half (*p̅*_1_ = *p̅*_2_ = Δ = ½). The average effect of an allele substitution (α) is reduced to the homozygote contrast, *a*. Substituting Equation 4 to obtain the generation means for an inbred line cross resulted in:

F_2_ = *μ* − ½*fd*

P_1_ = *μ* − ½*d* + *a*

P_2_ = *μ* − ½*d* − *a*

F_1_ = *μ* − ½ ( 1 − 2 )*d*

BC_P1_ = *μ* − ½*fd* + ½*a*

BC_P2_ = *μ* − ½*fd* − ½*a*

### Heterosis and inbreeding depression

**Figure 2:**
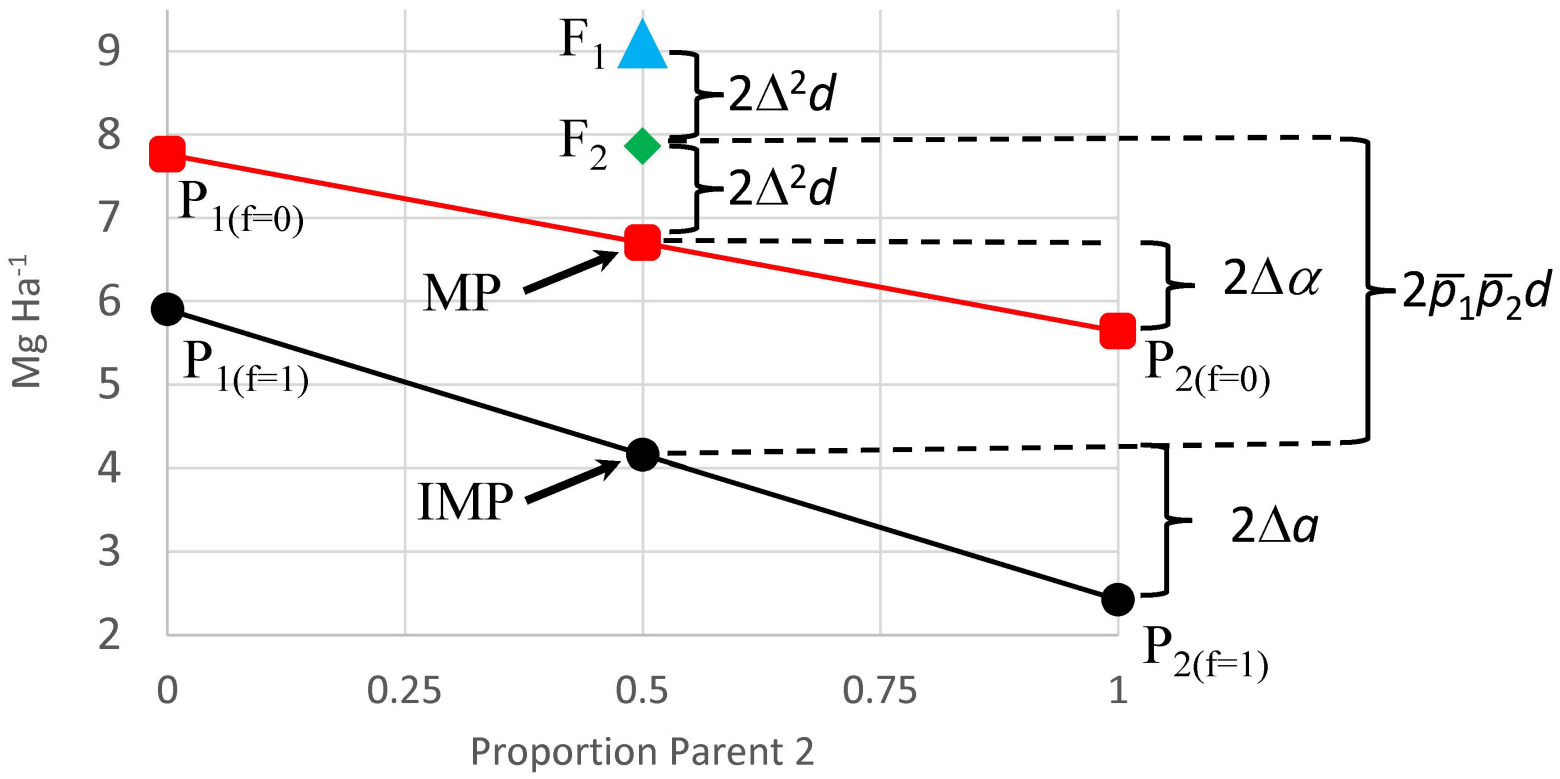
Schematic of generation means and estimators of heterosis. The top line (in red) represents values of P_1_, P_2_, and the midparent value (MP). The bottom line represents average values of inbred lines from P1 and P2 and the average of inbred lines across parensts (IMP). Values of F_2_ and F_1_ exceed miparent value by 2Δ^2^*d* and 4Δ^2^*d* respectively.

Midparent heterosis is defined as the difference between a crossbred generation and the average of the parents (Falconer and Mackay 1996). We defined four measures of heterosis (**Table 1**, **Figure 2**) starting with the base definition of midparent heterosis (MH) as the difference between the F_1_ cross and the mean of the parents, or midparent value (MP), which is 4Δ^2^*d* (**Table 1**, **Figure 2**). If the F_1_ generation is randomly mated for one generation to produce an F_2_ generation, heterosis is reduced by half to a value of 2Δ^2^*d* (**Table 1**, **Figure 2**). Inbreeding depression was incorporated by modeling the case in which parent populations were inbred to homozygosity to produce a population of inbred lines (**Figures 1**, **2**). Inbred midparent heterosis (IMH) is the difference between the F_1_ generation and inbred parents which has a value of 2*p̅*_1_*p̅*_2_d+2Δ^2^*d*. Random mating the F_1_ generation reduces inbred midparent heterosis by 2Δ^2^*d* to a value of 2*p̅*_1_*p̅*_2_d, which is equivalent to inbreeding depression in the F_2_ generation if it is inbred to homozygosity. Average inbreeding depression in the parents, 2*p̅*_1_*p̅*_2_d−2Δ^2^*d*, can be expressed in terms of parental allele frequencies by substituting parental allele frequencies for average frequencies and delta: 2*p̅*_1_*p̅*_2_*d*−2Δ^2^*d* = ½(*p*_1_ + *p*_1_’)(*p*_2_ + *p*_2_’)*d* – ½(*p*_1_−*p*_1_’)(*p*_2_−*p*_2_’)*d* = ½(*p*_1_*p*_2_ + *p*_1_’*p*_2_’)*d*. Because allele frequencies and Δ^2^ must be greater than or equal to zero, if *d* is positive, inbreeding depression is necessarily greater than or equal to zero and in addition *p̅*_1_*p̅*_2_≥Δ^2^. This inequality establishes two additional inequalities from expressions in **Table 1** which are i.) midparent value is always greater than or equal to inbred midparent value (MP ≥ IMP), and, ii.) inbred midparent heterosis is always greater than or equal to midparent heterosis (IMH ≥ MH).

**Table 1:** Predicted means for each generation.

F_2_	μ	μ
F_1_	μ + 2Δ^2^*d*	μ + ½*d*
F_1-selfed_	μ − *p̅*_1_*p̅*_2_*d* + Δ^2^*d*	μ
P_1 (f=0)_	μ − 2Δ^2^*d* + 2Δα	μ – ½*d* + *a*
P_2 (f=0)_	μ − 2Δ^2^*d* − 2Δα	μ – ½*d* – *a*
P_1 (f=1)_	μ − 2*p̅*_1_*p̅*_2_*d* + 2Δ*a*	μ – ½*d* + *a*
P_2 (f=1)_	μ − 2*p̅*_1_*p̅*_2_*d* − 2Δ*a*	μ – ½*d* – *a*
BC_P1_	μ+Δα	μ + ½*a*
BC_P2_	μ −Δα	μ – ½*a*
MP	μ −2Δ^2^*d*	μ – ½*d*
IMP	μ − 2*p̅*_1_*p̅*_2_*d*	μ – ½*d*
MH	4Δ^2^*d*	*d*
IMH	2*p̅*_1_*p̅*_2_*d* + 2Δ^2^*d*	*d*
MHF_2_	2Δ^2^*d*	½*d*
IMHF_2_	2*p̅*_1_*p̅*_2_*d*	½*d*
ID	2*p̅*_1_*p̅*_2_*d* – 2Δ^2^*d* =½(2*p*_1_*p*_2_*d* +2*p*_1_’*p*_2_’*d*)	0

MP, midparent value at panmixia; IMP, inbred midparent; MH, midparent heterosis; IMH, inbred-midparent heterosis; MHF_2_, midparent F_2_ heterosis; IMHF_2_, inbred-midparent F_2_ heterosis; ID, inbreeding depression in parents. Variables μ, a, d, *p̅*_1_, *p̅*_2_, and Δ are defined in *Materials and methods*.

The theory proposed by Wilham and Pollak (1985) extended by Lamkey and Edwards (1999) and in the present study was based on two parental populations but does not necessarily include any assumptions with respect to the origin of the parental populations. An important special case of the theory is the case in which parent populations diverge from a common base population due to genetic drift. The degree of divergence of subpopulations can be quantified using Wright’s *F*_ST_ (Wright, 1951), which quantifies increased identity by descent within a subpopulation relative to the between-population identity. If a population is divided arbitrarily into two parental populations, each with a large sample of individuals, *F*_ST_ and Δ are both equal to zero. With Δ = 0, midparent heterosis equals zero, and inbred midparent heterosis is equal to inbreeding depression in the base population, 2*p̅*_1_*p̅*_2_*d*. At the other extreme, that is, when *F*_ST_ = 1, if different alleles were fixed in the two parents, δ is at a maximum value of 0.5, inbreeding depression is zero (because parents are inbred to homozygosity and cannot be inbred further), and midparent heterosis is equal to inbred midparent heterosis, both equaling a value of *d*.

For intermediate values of Δ corresponding to 0 <*F*_ST_*<*1, as Δ increases from zero to a maximum of 0.5, midparent heterosis increases, whereas inbreeding depression (the reduction in value when parents are inbred) and midparent value decrease. Because heterosis increases with Δ and midparent value and inbreeding depression decrease, a negative correlation between heterosis and midparent value and between heterosis and inbreeding depression is implied. This theory illustrates that inbreeding depression is an important surrogate measure of population divergence under the drift model. Low inbreeding depression within parent populations suggests that populations have already undergone significant inbreeding and divergence from one another, indicating high Δ and heterosis. High inbreeding depression within parent populations suggests the opposite; populations are still highly heterozygous, and Δ is low. Because high heterosis is associated with high Δ, it is also associated with a low midparent value. Hence, selection for high heterosis may be more likely to result in a low parent value than in a high F_1_-performance.

The present theory, under divergence due to drift, can be related directly to heterozygosity to further illustrate the relationship between inbreeding depression and heterosis. Heterozygosity in the F_2_ reference population is equal to 2*p̅*_1_*p̅*_2_. Heterozygosity in the F_1_ is 2*p̅*_1_*p̅*_2_+2Δ^2^ and average heterozygosity in parent populations is 2*p̅*_1_*p̅*_2_−2Δ^2^, showing that 2Δ^2^ is the deviation in heterozygosity of F_1_ and parental generations from the F_2_ generation. Mean values of the F_2_, F_1_, and parents (MP) are *μ*, *μ* + 2Δ^2^*d*, *μ* - 2Δ^2^*d*, showing that the F_1_ exceeds the F_2_ by 2Δ^2^*d* and F_2_ exceeds parents by the same quantity, 2Δ^2^*d*, the difference in heterozygosity times dominance. If the inbreeding of the parental populations relative to the F_2_ reference population is represented by *F*_ST_, the average heterozygosity of parent populations, *H*_P_, is equal to heterozygosity in the F_2_ generation (*H*_F2_) times 1-*F*_ST_, i.e., *H*_P_ = (1-*F*_ST_)*H*_F2_. Substituting heterozygosity in terms of average allele frequencies and delta and rearranging results in an expression for delta of the form 2Δ^2^ = 2*F*_ST_*p̅*_1_*p̅*_2_, which shows that under a model divergence due to drift, expected divergence in allele frequencies is a function of *F*_ST_, with the important caveat that this will be an average over many potential parent populations undergoing inbreeding. For any two particular parent populations undergoing inbreeding and divergence, random drift of allele frequencies will generate random deviations from the exact relationship between Δ^2^ and *F*_ST_.

### Experimental treatments

The theory was tested empirically using a maize experiment containing crosses representing a wide range of parental genetic divergence. Six synthetic maize populations and eight inbred lines were used as the parents (**Table 2**). All populations were derived from three different synthetics: BSSS, BSCB1, and BS11. Synthetic BS13(S)C10 is a direct descendant of the BSSS synthetic population (**Table 2**). Both B129 and B73 inbred lines are members of the stiff stalk heterotic group, whereas the remaining inbred lines are members of the non-stiff stalk heterotic group. Three crosses were made between synthetic populations, three between synthetic populations and inbred line B129, and six between the inbred lines (**Table 3**). For each of the 12 crosses listed in **Table 3**, seeds were produced for parents (P_1_ and P_2_), F_1_ hybrid, self-pollinated F_1_ (F_1-selfed_), randomly mated F_1_ (F_2_), and backcrosses to parents 1 (BC_P1_) and 2 (BC_P2_), for a total of 60 pedigrees derived from crosses. Each of the six synthetic parent populations was self-pollinated for one generation to produce the S_1_ generation. Thus, a total of 80 pedigrees corresponding to 14 parents, six S_1_, and 60 cross-derived pedigrees were used in the experiment.

**Table 2:** Derivation process and development of the 14 parents evaluated in the experiment.

Pedigree	Derivation	Reference
BSSS(R)C15	The fifteenth cycle of reciprocal recurrent selection with BSCB1 as a tester in the selection scheme	Penny and Eberhart (1971)
BSCB1(R)C15	The fifteenth cycle of reciprocal recurrent selection with BSSS as a tester in the selection scheme	Penny and Eberhart (1971)
BSCB1(R)C16	The sixteenth cycle of reciprocal recurrent selection with BSSS as a tester in the selection scheme	Penny and Eberhart (1971)
BS13(S)C10	The tenth cycle of an S_2_-progeny selection from the recombination of 29 S_1_ lines	Lamkey (1992)
BS11(FR)C15	The fifteenth cycle of a reciprocal full-sib recurrent selection (FR) between BS11 and BS10	Weyhrich et al. (1998)
BSKRL4(HI)C2	The second cycle of a half-sib recurrent selection program started at Iowa State University	
B114	Line developed based on testcross performance from “Pool 41-C15-19-2-1-1-1-1-1-1” with the tester A632	Hallauer et al. (2000)
B116	Line from the sixth generation of pedigree selection applied to the B97/B99 F_2_ population	Hallauer et al. (2004)
B129	Line produced by single seed descent (SSD) from the cross B73/B84	
B73	Line selected in the fifth cycle of the reciprocal recurrent selection with BSCB1 as a common tester	Russell (1972)
BX010	An S_12_ experimental line developed from the cross B95/B97	
Mo17	Line selected from the single cross CI187-2/C103 in the Missouri Agricultural Experiment Station	Zuber (1973)
SGI912	Line developed by Seed Genetics Inc. from the cross B73/B37	
TR7245	Line developed by Thurston Genetics	

**Table 3:** Crosses made between the synthetic populations, between synthetic populations and the inbred line B129, and between inbred lines.

Cross	Parent 1	Parent 2
1	BS13(S)C10	BSCB1(R)C15
2	BS13(S)C10	BSSS(R)C15
3	BSSS(R)C15	BSCB1(R)C15
4	BS11(FR)C15	B129
5	BSCB1(R)C16	B129
6	BSKRL4(HI)C2	B129
7	B129	B114
8	B129	B116
9	B73	Mo17
10	SGI912	BX010
11	SGI912	B116
12	TR7245	B116

### Experimental design

The 80 pedigrees were grown in five locations near Ames, Carroll, Crawfordsville, Fairfield, and Lewis, Iowa, during the growing seasons of 2007 and 2008. Sixteen of the 80 pedigrees were replicated multiple times within each replicate block in each location, resulting in 100 experimental units per replicate. The experiment was arranged in a modified split-plot design with three replications, where the inbreeding level (F_1_, F_2_, backcrosses, inbred lines, and synthetic populations) was considered as the whole-plot treatment factor and pedigree within the inbreeding level as the subplot treatment factor. The design was described as a modified split plot because subplots (pedigrees) were not cross-classified with whole plots and because each level of the whole plot factor was applied to more than one whole-plot experimental unit within a replicate to reduce the size of whole-plot experimental units. The experimental unit for the subplot factor was a plot of four rows, spaced 0.76 m apart and 5.49 m in length. The whole-plot experimental unit was a block containing five subplots side-by-side (20 rows, 5.49 m long), with one of five possible inbreeding levels applied to each whole-plot experimental unit. Each replicate containing 100 subplots was divided into 20 whole-plot blocks, which were then separated into two, three, three, five, and seven blocks for the synthetic populations, inbred lines, F_1_, backcrosses, and F_2_ (including the selfed F_1_) inbreeding levels, respectively. Each whole-plot experimental unit was randomly assigned to a range in the field. Within each replicate block, there were 10, 15, 15, 25, and 35 subplots (pedigrees) for synthetic populations, inbred lines, F_1_, backcrosses, and F_2_, respectively. The seeds were sown at a density of 7.0 plants per m^2^ using an Almaco mini-belt cone planter. Agronomic practices were applied to each experiment, following commercial maize production practices in central Iowa. The sampling unit consisted of two central rows of each plot to avoid border effects.

### Data collection

Data were collected on a plot basis in the two center rows for plant height (cm), ear height (cm), and grain yield (Mg ha^−1^) adjusted to 15.5% grain moisture. Plant height (cm) and ear height (cm) were recorded during 2007 and 2008 for the experiments grown near Carroll and Lewis, and during 2007 for the experiments grown near Crawfordsville. Plant height was measured as the distance from the soil surface to the flag leaf collar. Similarly, the ear height was measured as the distance from the soil surface to the uppermost ear node. The mean values for plant and ear heights were recorded as the average of 10 randomly selected plants per plot. Plots were harvested using a New Holland TR88 combine modified for the automatic acquisition of test weight, grain moisture, and grain weight. An accurate grain moisture measurement requires a minimum of approximately 1.8 kg. The location Fairfield, 2007, had 20% of plots with grain mass less than 1.8 kg and thus, was excluded from the analysis for grain yield.

### Data analysis

Data were analyzed by fitting the linear mixed-effects model:


$$Y_{ijklmn}=\xi_{i}+\Gamma_{l(i)}+I_{j}+\omega_{ij}+\psi_{j(k)}+\phi_{ik(j)}+v_{m(il)}+\delta_{n(il)}+\epsilon_{ijklmn}$$

Where *Y_ijklmn_* was the response variable for range *n*, pass *m*, replication *l*, pedigree *k*, inbreeding level *j*, environment *i*, ξ*_i_* effect of environment *i*, Γ*_l_*_(_*_i_*_)_ replication *l* within environment *i*, *I_j_* inbreeding level *j*, ω*_ij_* interaction between environment *i* and inbreeding level *j*, ψ*_j_*_(_*_k_*_)_ pedigree *k* within inbreeding level *j*, ϕ*_ik_*_(_*_j_*_)_ interaction between pedigree *k* and environment *i* within inbreeding level *j*, ν*_m_*_(_*_il_*_)_ pass *m* within replication *l* and environment *i*, δ*_n_*_(_*_il_*_)_ range *n* within replication *l* and environment *i*, and ε*_ijklmn_* was residual error. All effects were fit as fixed effects except the environmental interactions ω*_ij_* and ϕ*_ik_*_(_*_j_*_)_, and range within replication and environment, δ*_n_*_(_*_il_*_)_. Outliers were identified by fitting the full linear model and estimating the probability of obtaining a larger absolute value for each residual using a t-distribution and adjusting individual p-values with a Bonferroni correction at a 2% level of significance. After removing outliers, variances of residuals (ε*_ijklmn_*) and the range within replication and environment (δ*_n_*_(_*_il_*_)_) were considered to be heterogeneous among environments such that V(ε*_ijklmn_*) = σ^2^*_e_*_(_*_i_*_)_ and V(δ*_n_*_(_*_il_*_)_) =σ^2^*_δ_*_(_*_i_*_)_. Twenty-four models were generated from all possible combinations of including or excluding each of the three random effects (environmental interaction and random range effects) in the model and including or excluding heterogeneity of residual and range-block variances (V(ε*_ijklmn_*) = σ^2^*_e_* vs. V(ε*_ijklmn_*) = σ^2^*_e_*_(_*_i_*_)_ V(δ*_n_*_(_*_il_*_)_) =σ^2^*_δ_* vs. V(δ*_n_*_(_*_il_*_)_) = σ^2^*_δ_*_(_*_i_*_)_) (**Table 4**). The Bayesian Information Criterion (BIC) was obtained from fitting each of the 24 models and the best model chosen from lowest BIC (Schwarz, 1978). Residual Maximum Likelihood (REML) was used to obtain the estimates of the variances by using the Fisher-Scoring algorithm in ASReml (Gilmour et al., 2015). These estimates were used to fit a mixed model in proc mixed in SAS software (SAS Institute, Cary, NC) to compute tests of fixed effects and best linear unbiased estimators (BLUEs) for environments, inbreeding levels, and pedigrees within inbreeding level. All analyses were done using the MIXED procedure of SAS 9.4 software (SAS Institute, Cary, NC) and ASReml release 4.1 software (Gilmour et al., 2015).

**Table 4:** Bayesian information criteria (BIC) for 24 possible linear models.

Env × Inb	Env × Ped (Inb)	Range (Rep × Env)	Het Error †	Het Range ‡	Grain Yield	Plant Height	Ear Height
X	X	X	N	Y	3,298.6	7,541.7	6,736.1
X	X	X	N	N	3,342.4	7,563.2	6,716.2
X	X	X	Y	N	3,186.6	7,482.8	6,702.3
X	X	X	Y	Y	3,181.2*	7,460.8	6,729.3
X	X		N	N	3,462.3	7,675.4	6,736.0
X	X		Y	N	3,245.6	7,594.6	6,721.9
X		X	N	Y	3,434.7	7,595.1	6,764.6
X		X	N	N	3,458.0	7,614.5	6,746.1
X		X	Y	N	3,285.1	7,556.8	6,726.2
X		X	Y	Y	3,280.7	7,532.9	6,752.3
	X	X	N	Y	3,308.1	7,534.5	6,729.2
	X	X	N	N	3,352.9	7,556.0	6,709.3
	X	X	Y	N	3,200.2	7,475.6	6,695.6*
	X	X	Y	Y	3,194.0	7,453.7*	6,722.6
X			N	N	3,547.1	7,718.3	6,762.0
X			Y	N	3,338.3	7,661.0	6,742.2
	X		N	N	3,495.1	7,668.8	6,730.0
	X		Y	N	3,273.0	7,587.4	6,716.4
		X	N	Y	3,462.1	7,587.9	6,759.1
		X	N	N	3,488.5	7,607.3	6,740.0
		X	Y	N	3,315.5	7,549.7	6,721.0
		X	Y	Y	3,311.3	7,525.8	6,746.8
			N	N	3,649.7	7,717.2	6,759.4
			Y	N	3,415.7	7,660.3	6,742.0

Models contained all fixed effects described in the materials and methods section plus random effects containing an ‘X’ in the corresponding column. Lowest BIC value for each model is denoted with an asterisk. Env, environment; Rep, replication; Ped, pedigree; Inb, inbreeding level. The Het Error and Het Range columns indicate whether error variances and range variances were fit as heterogeneous among environments with Y indicating heterogeneous.

### Genetic parameter estimation

The generations evaluated in this experiment and listed in **Table 1** were represented by five genetic parameters, *μ*, Δ^2^*d*, *p̅*_1_*p̅*_2_*d*, Δ*a*, and Δ*α* for population crosses (including population by inbred crosses) and three parameters, *μ*, *a*, and *d*, for inbred-by-inbred crosses (**Table 1**). Genetic parameters were estimated with weighted least squares (WLS) regression using BLUEs of generation means from the linear mixed-effects model fit to raw phenotypic data from the multi-environment trial. For each cross, there were BLUEs for 7 generations which were F_1_, F_2_, F_1-selfed_, P_1_, P_2_, BC_1_, and BC_2_. In addition, BLUEs were fit in the model for self-pollinated synthetic population crosses (*f*=0.5). A full model containing all parameters was fit to generation means and subsequently non-significant parameters were removed from the model until all parameters retained were significant (Christensen, 2011).

## Results

Based on Bonferroni adjusted p-values (data not shown), twenty-two, thirteen, and fourteen observations were identified as outliers and removed from the analysis for grain yield, plant height, and ear height, respectively. Bayesian information criterion (BIC) is given for each of the 24 fitted models in **Table 4**. The best-fitting model contained heterogeneous residual variances among environments (V(ε*_ijklm_*) = σ^2^*_e_*_(_*_i_*_)_) for all traits (**Table 4**) and heterogeneity among environments of the variance of range effects (δ*_n_*_(_*_il_*_)_) for all traits except ear height (**Table 4**). For grain yield, a model including all random effects was selected, while for the other two traits a model excluding the environment by inbreeding level interaction effect (ω*_ij_*) was the best fitting model (**Table 4**).

### Model testing

For all crosses and traits, the genetic parameters μ, Δ^2^*d*, and *p̅*_1_*p̅*_2_*d*, were significant whereas additive parameters Δ*a* and Δα were not significant in some cross by trait combinations (**Tables 5**, **6**). Midparent heterosis (MH) and inbred-midparent heterosis (IMH), calculated from the genetic parameters in **Tables 5**, **6**, were significantly different from zero for all traits and crosses (**Table 7**). For inbred line crosses, midparent and inbred-midparent heterosis were equivalent because inbred lines cannot be inbred further, so inbreeding depression is zero. For crosses involving a panmictic population, inbred-midparent heterosis was significantly higher than MH for all crosses and traits (**Table 7**) as predicted by the model.

**Table 5:** Estimates of the model parameters μ, Δ^2^*d*, Δα, Δ*a*, and *p̅*_1_*p̅*_2_*d* in the population-by-population (PxP) and population-by-inbred (PxI) crosses for grain yield (Mg ha^-1^), ear height (cm), and plant height (cm).

Pedigree	Cross	μ	Δ^2^*d*	Δ*a*	Δα	*p̅*_1_ *p̅*_2_ *d*
Grain yield (Mg ha^-1^)						
BS11(FR)C15/B129	PxI	7.84 ± 0.26	0.82 ± 0.19			1.93 ± 0.27
BSCB1(R)C16/B129	PxI	7.50 ± 0.08	1.07 ± 0.07	-0.56 ± 0.09	0.27 ± 0.09	2.13 ± 0.10
BSKRL4(HI)C2/B129	PxI	7.24 ± 0.11	1.31 ± 0.08	-0.59 ± 0.14		2.04 ± 0.16
BS13(S)C10/BSCB1(R)C15	PxP	7.86 ± 0.19	0.58 ± 0.12	0.87 ± 0.33	0.53 ± 0.14	1.85 ± 0.31
BS13(S)C10/BSSS(R)C15	PxP	8.04 ± 0.07	0.40 ± 0.05	0.70 ± 0.12	0.31 ± 0.05	1.71 ± 0.12
BSSS(R)C15/BSCB1(R)C15	PxP	7.41 ± 0.08	0.65 ± 0.05		0.31 ± 0.05	2.25 ± 0.13
Ear height (cm)						
BS11(FR)C15/B129	PxI	109.40 ± 1.22	1.17 ± 0.90			11.01 ± 1.14
BSCB1(R)C16/B129	PxI	96.44 ± 0.82	4.08 ± 0.69	-5.44 ± 0.86	-3.16 ± 0.92	10.31 ± 0.98
BSKRL4(HI)C2/B129	PxI	102.49 ± 0.67	5.13 ± 0.47	-2.38 ± 0.81		10.29 ± 0.92
BS13(S)C10/BSCB1(R)C15	PxP	92.67 ± 0.69	4.01 ± 0.42	3.19 ± 1.08	1.12 ± 0.46	9.06 ± 1.08
BS13(S)C10/BSSS(R)C15	PxP	99.06 ± 0.73	2.90 ± 0.44		-2.68 ± 0.44	9.98 ± 1.13
BSSS(R)C15/BSCB1(R)C15	PxP	98.32 ± 1.87	3.62 ± 1.14		5.23 ± 1.14	11.13 ± 2.90
Plant height (cm)						
BS11(FR)C15/B129	PxI	219.80 ± 1.75	3.96 ± 1.48		6.76 ± 1.95	18.39 ± 1.54
BSCB1(R)C16/B129	PxI	207.48 ± 2.39	9.11 ± 1.74	-6.30 ± 2.82		18.41 ± 3.21
BSKRL4(HI)C2/B129	PxI	218.10 ± 1.27	6.77 ± 0.91			17.42 ± 1.28
BS13(S)C10/BSCB1(R)C15	PxP	206.11 ± 1.53	9.09 ± 0.94		-2.18 ± 0.96	19.87 ± 2.42
BS13(S)C10/BSSS(R)C15	PxP	210.30 ± 0.93	5.14 ± 0.57	-4.47 ± 1.51	-7.81 ± 0.64	17.34 ± 1.47
BSSS(R)C15/BSCB1(R)C15	PxP	217.56 ± 2.62	6.52 ± 1.62		7.62 ± 1.65	20.75 ± 4.14

Parameter were defined in the methods section.

**Table 6:** Estimates of the genetic parameters *m*, *d*, and *a* for grain yield (Mg ha^−1^), ear height (cm), and plant height (cm) in the inbred-by-inbred crossed.

Cross	*M*	*d*	*a*
Grain yield (Mg ha^−1^)			
B129/B114	3.46 ± 0.20	7.11 ± 0.35	0.95 ± 0.20
B129/B116	4.13 ± 0.34	6.61 ± 0.66	
B73/Mo17	4.36 ± 0.39	4.51 ± 0.70	
SGI912/BX010	4.64 ± 0.41	5.72 ± 0.77	1.32 ± 0.41
SGI912/B116	4.68 ± 0.21	6.08 ± 0.39	1.23 ± 0.21
TR7245/B116	4.84 ± 0.21	5.63 ± 0.38	1.30 ± 0.21
Ear Height (cm)			
B129/B114	76.58 ± 0.96	29.97 ± 1.77	9.50 ± 0.98
B129/B116	85.54 ± 1.68	25.30 ± 3.52	
B73/Mo17	89.66 ± 1.45	27.61 ± 2.55	6.00 ± 1.49
SGI912/BX010	86.96 ± 0.73	31.34 ± 1.44	−3.32 ± 0.73
SGI912/B116	85.25 ± 1.22	29.12 ± 2.40	
TR7245/B116	90.53 ± 1.43	24.99 ± 2.62	6.01 ± 1.44
Plant Height (cm)			
B129/B114	171.47 ± 2.39	54.00 ± 4.43	10.66 ± 2.44
B129/B116	195.04 ± 4.23	41.37 ± 8.61	−13.91 ± 4.24
B73/Mo17	186.04 ± 1.11	49.27 ± 1.98	5.05 ± 1.14
SGI912/BX010	184.91 ± 1.20	63.96 ± 2.36	−8.39 ± 1.20
SGI912/B116	192.19 ± 1.15	58.06 ± 2.23	−16.93 ± 1.16
TR7245/B116	196.81 ± 1.66	46.93 ± 3.06	−12.09 ± 1.67

Parameters were defined in the methods section.

**Table 7:** Model-based predictions of the mean (and standard error) for midparent value (MP), inbred midparent value (IMP), midparent heterosis (MH), inbred-midparent heterosis (IMH), midparent F_2_ (MF_2_H) heterosis, and inbred-midparent F_2_ (IMF_2_H) heterosis for grain yield (Mg ha^−1^), ear height (cm), and plant height (cm) for the inbred-by-inbred (I × I), population-by-inbred (P × I), and population-by-population (P × P) crosses.

Pedigree	Cross	MP	IMP	MH	IMH	MF_2_H	IMF_2_H
Grain yield (Mg ha^−1^)							
B129/B114	I × I	3.46 ± 0.20	3.46 ± 0.20	7.11 ± 0.35	7.11 ± 0.35	3.55 ± 0.18	3.55 ± 0.18
B129/B116	I × I	4.13 ± 0.34	4.13 ± 0.34	6.61 ± 0.66	6.61 ± 0.66	3.31 ± 0.33	3.31 ± 0.33
B73/Mo17	I × I	4.36 ± 0.39	4.36 ± 0.39	4.51 ± 0.70	4.51 ± 0.70	2.26 ± 0.35	2.26 ± 0.35
SGI912/BX010	I × I	4.64 ± 0.41	4.64 ± 0.41	5.72 ± 0.77	5.72 ± 0.77	2.86 ± 0.39	2.86 ± 0.39
SGI912/B116	I × I	4.68 ± 0.21	4.68 ± 0.21	6.08 ± 0.39	6.08 ± 0.39	3.04 ± 0.19	3.04 ± 0.19
TR7245/B116	I × I	4.84 ± 0.21	4.84 ± 0.21	5.63 ± 0.38	5.63 ± 0.38	2.81 ± 0.19	2.81 ± 0.19
BS11(FR)C15/B129	P × I	6.20 ± 0.47	3.97 ± 0.44	3.28 ± 0.78	5.50 ± 0.67	1.64 ± 0.39	3.87 ± 0.55
BSCB1(R)C16/B129	P × I	5.36 ± 0.18	3.24 ± 0.17	4.28 ± 0.28	6.41 ± 0.23	2.14 ± 0.14	4.27 ± 0.20
BSKRL4(HI)C2/B129	P × I	4.61 ± 0.20	3.15 ± 0.27	5.26 ± 0.32	6.72 ± 0.34	2.63 ± 0.16	4.09 ± 0.32
BS13(S)C10/BSCB1(R)C15	P × P	6.70 ± 0.26	4.17 ± 0.54	2.31 ± 0.48	4.85 ± 0.67	1.15 ± 0.24	3.69 ± 0.62
BS13(S)C10/BSSS(R)C15	P × P	7.24 ± 0.10	4.63 ± 0.20	1.60 ± 0.18	4.21 ± 0.25	0.80 ± 0.09	3.41 ± 0.23
BSSS(R)C15/BSCB1(R)C15	P × P	6.12 ± 0.11	2.92 ± 0.22	2.58 ± 0.19	5.78 ± 0.27	1.29 ± 0.10	4.49 ± 0.25
Ear height (cm)							
B129/B114	I × I	76.58 ± 0.96	76.58 ± 0.96	29.97 ± 1.77	29.97 ± 1.77	14.98 ± 0.89	14.98 ± 0.89
B129/B116	I × I	85.54 ± 1.68	85.54 ± 1.68	25.30 ± 3.52	25.30 ± 3.52	12.65 ± 1.76	12.65 ± 1.76
B73/Mo17	I × I	89.66 ± 1.45	89.66 ± 1.45	27.61 ± 2.55	27.61 ± 2.55	13.80 ± 1.28	13.80 ± 1.28
SGI912/BX010	I × I	86.96 ± 0.73	86.96 ± 0.73	31.34 ± 1.44	31.34 ± 1.44	15.67 ± 0.72	15.67 ± 0.72
SGI912/B116	I × I	85.25 ± 1.22	85.25 ± 1.22	29.12 ± 2.40	29.12 ± 2.40	14.56 ± 1.20	14.56 ± 1.20
TR7245/B116	I × I	90.53 ± 1.43	90.53 ± 1.43	24.99 ± 2.62	24.99 ± 2.62	12.49 ± 1.31	12.49 ± 1.31
BS11(FR)C15/B129	P × I	107.07 ± 2.18	87.37 ± 1.76	4.66 ± 3.58	24.36 ± 2.88	2.33 ± 1.79	22.03 ± 2.27
BSCB1(R)C16/B129	P × I	88.28 ± 1.81	75.82 ± 1.65	16.33 ± 2.75	28.79 ± 2.27	8.17 ± 1.38	20.62 ± 1.95
BSKRL4(HI)C2/B129	P × I	92.23 ± 1.11	81.91 ± 1.54	20.51 ± 1.86	30.83 ± 2.02	10.25 ± 0.93	20.57 ± 1.83
BS13(S)C10/BSCB1(R)C15	P × P	84.65 ± 0.89	74.54 ± 1.85	16.05 ± 1.70	26.16 ± 2.32	8.03 ± 0.85	18.13 ± 2.15
BS13(S)C10/BSSS(R)C15	P × P	93.26 ± 0.94	79.10 ± 1.94	11.60 ± 1.77	25.76 ± 2.46	5.80 ± 0.88	19.96 ± 2.27
BSSS(R)C15/BSCB1(R)C15	P × P	91.08 ± 2.42	76.05 ± 4.97	14.49 ± 4.56	29.51 ± 6.28	7.24 ± 2.28	22.27 ± 5.81
Pedigree	Cross	MP	IMP	MH	IMH	MF_2_H	IMF_2_H
Plant height (cm)							
B129/B114	I × I	171.47 ± 2.39	171.47 ± 2.39	54.00 ± 4.43	54.00 ± 4.43	27.00 ± 2.21	27.00 ± 2.21
B129/B116	I × I	195.04 ± 4.23	195.04 ± 4.23	41.37 ± 8.61	41.37 ± 8.61	20.69 ± 4.30	20.69 ± 4.30
B73/Mo17	I × I	186.04 ± 1.11	186.04 ± 1.11	49.27 ± 1.98	49.27 ± 1.98	24.64 ± 0.99	24.64 ± 0.99
SGI912/BX010	I × I	184.91 ± 1.20	184.91 ± 1.20	63.96 ± 2.36	63.96 ± 2.36	31.98 ± 1.18	31.98 ± 1.18
SGI912/B116	I × I	192.19 ± 1.15	192.19 ± 1.15	58.06 ± 2.23	58.06 ± 2.23	29.03 ± 1.12	29.03 ± 1.12
TR7245/B116	I × I	196.81 ± 1.66	196.81 ± 1.66	46.93 ± 3.06	46.93 ± 3.06	23.47 ± 1.53	23.47 ± 1.53
BS11(FR)C15/B129	P × I	211.89 ± 3.89	183.02 ± 2.41	15.82 ± 5.94	44.69 ± 4.00	7.91 ± 2.97	36.78 ± 3.08
BSCB1(R)C16/B129	P × I	189.26 ± 4.30	170.66 ± 5.34	36.44 ± 6.95	55.04 ± 6.90	18.22 ± 3.47	36.82 ± 6.42
BSKRL4(HI)C2/B129	P × I	204.56 ± 2.13	183.26 ± 2.05	27.09 ± 3.63	48.39 ± 3.20	13.54 ± 1.81	34.85 ± 2.56
BS13(S)C10/BSCB1(R)C15	P × P	187.93 ± 2.04	166.38 ± 4.14	36.36 ± 3.76	57.92 ± 5.21	18.18 ± 1.88	39.74 ± 4.83
BS13(S)C10/BSSS(R)C15	P × P	200.02 ± 1.24	175.61 ± 2.52	20.56 ± 2.29	44.96 ± 3.17	10.28 ± 1.14	34.68 ± 2.94
BSSS(R)C15/BSCB1(R)C15	P × P	204.53 ± 3.49	176.06 ± 7.09	26.07 ± 6.46	54.54 ± 8.90	13.03 ± 3.23	41.51 ± 8.27

Predictions were obtained from expressions in **Table 1**.

**Figure 3:**
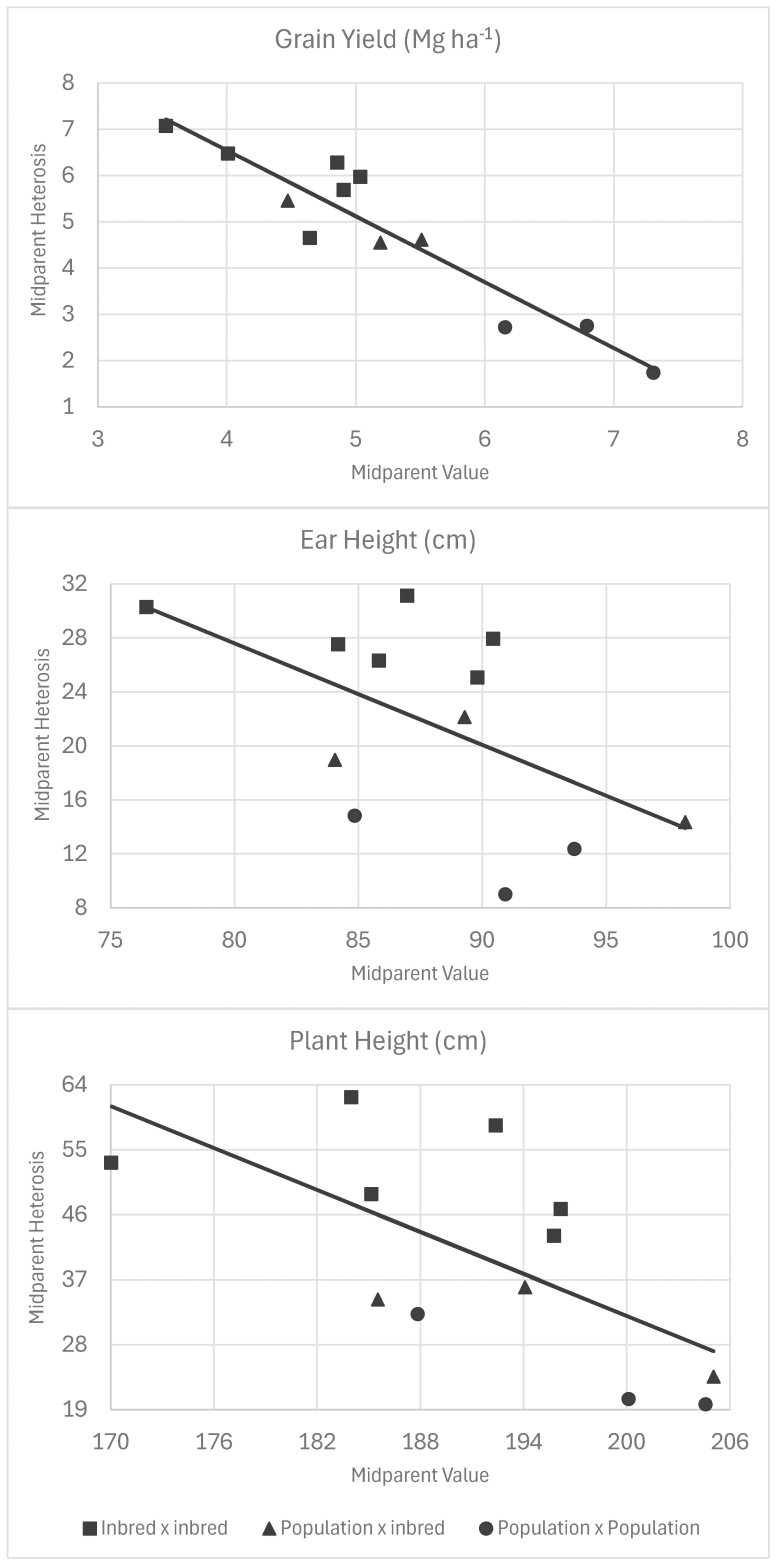
Simple linear regression of midparent heterosis versus midparent values for all crosses for grain yield, plant height, and ear height. The dots correspond to inbred-by-inbred crosses, triangles to population-by-inbred crosses, and squares to population-by-population crosses.

**Figure 4:**
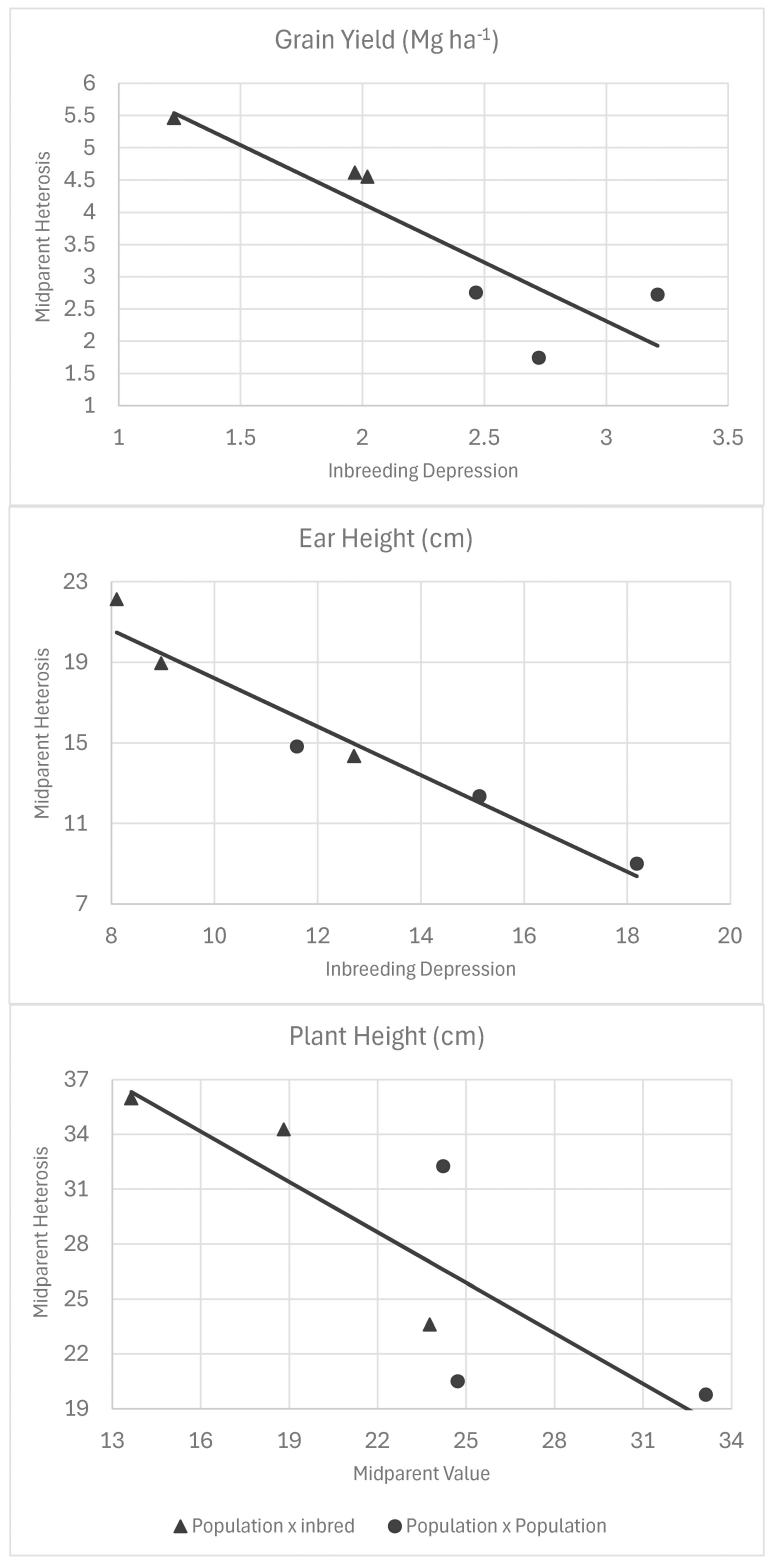
Simple linear regression of midparent heterosis versus inbreeding depression in the parent population for grain yield, plant height, and ear height. Triangles represent population-by-inbred crosses, and dots represent population-by-population crosses.

The theory predicts that midparent heterosis is negatively related to both midparent value and inbreeding depression in parents. Our study included crosses that covered a wide range of inbreeding levels, and hence, the degree of parental divergence (2Δ^2^) and midparent value. For grain yield, midparent values predicted 86% of the variation in midparent heterosis based on the regression of midparent heterosis onto midparent value (**Figure 3**, **Table 8**). For every increase of 1.0 Mg ha^−1^ in midparent grain yield, midparent heterosis decreased by 1.42 Mg ha^−1^ (**Figure 3**). Inbreeding depression in the parents predicted 70% of midparent heterosis in the regression of midparent heterosis onto inbreeding depression, with a decrease of 1.82 Mg ha^−1^ in midparent heterosis for grain yield per 1.0 Mg ha^−1^ in inbreeding depression (**Table 8**). It should be noted that the regression of heterosis onto midparent value included all crosses, whereas the regression onto inbreeding depression did not include inbred-by-inbred crosses because inbreeding depression is zero in inbred-by-inbred crosses. For plant height and ear height, the same relationship was observed, but the midparent value was much less predictive of heterosis than it was for grain yield, with R^2^ values of 38% for plant height and 23% for ear height (compared to 86% for grain yield) (**Figure 3**). Inbreeding depression for plant height predicted 60% of mid-parent heterosis and 93% of mid-parent heterosis for ear height (**Figure 4**).

**Table 8:** Regression parameters for regression of midparent heterosis onto midparent value and inbreeding depression of parent populations.

Predictor	Trait	Intercept	slope	R^2^
Midparent	Grain yield	12.2	−1.42	86.4
Midparent	Ear height	87.7	−0.75	23.4
Midparent	Plant height	225.6	−0.97	38.3
Inbreeding depression	Grain yield	7.8	−1.82	70.3
Inbreeding depression	Ear height	30.2	−1.20	93.2
Inbreeding depression	Plant height	48.9	−0.92	59.9

### Genetic importance of hybrid seed production

Over the past 50 to 70 years, maize production has been dominated by the use of single-cross hybrids. Farmer-saved seed from single-cross hybrids is the equivalent of the F_2_ generation and is well known to suffer a performance loss compared to the F_1_ generation (Hallauer and Miranda, 1988; Meghji et al., 1984). The predicted loss in performance is equivalent to half of the heterosis observed in the F_1_ or 2Δ^2^*d* (Falconer and Mackay, 1996; Lamkey and Edwards, 1999). The model also demonstrated that the loss in performance is a function of genetic divergence between parents, which is consistent with past observations that the reduction in performance in the F_2_ is smaller when open-pollinated varieties are used as parents than when inbred lines are used. The observed loss in performance was consistent with the model prediction for grain yield in our empirical study in eight of the 12 cases and differed from the prediction in the remaining four cases (**Table 9**).

**Table 9:** Mean values (and standard error) for the loss in the hybrid seed production system by saving seed from F_1_ hybrids for grain yield (Mg ha^−1^).

Cross	Grain yield (Mg ha^−1^)	
	Loss	Half MH
B129/B114	3.78 ± 0.38	3.54 ± 0.17
B129/B116	2.23 ± 0.39*	3.24 ± 0.17
B73/Mo17	3.31 ± 0.37*	2.33 ± 0.17
SGI912/BX010	4.07 ± 0.40*	2.99 ± 0.18
SGI912/B116	3.47 ± 0.38	3.14 ± 0.16
TR7245/B116	3.35 ± 0.37	2.84 ± 0.16
BS11(FR)C15/B129	2.44 ± 0.39	2.31 ± 0.17
BSCB1(R)C16/B129	2.43 ± 0.39	2.28 ± 0.17
BSKRL4(HI)C2/B129	2.86 ± 0.39	2.73 ± 0.16
BS13(S)C10/BSCB1(R)C15	2.19 ± 0.39*	1.38 ± 0.17
BS13(S)C10/BSSS(R)C15	1.24 ± 0.40	0.87 ± 0.17
BSSS(R)C15/BSCB1(R)C15	1.44 ± 0.39	1.36 ± 0.17

The loss was quantified as the difference between the F_1_ and F_2_ generation and was compared the model prediction of half midparent heterosis (2δd, MH). Significant differences (P <0.05) between yield loss and half midparent heterosis (Half MH) are indicated with an asterisk.

## Discussion

Past theory and extensions presented here to address inbreeding show that in a single-locus model with divergence due to drift, heterosis is a linear function of heterozygosity and directional dominance. The linear relationship with heterozygosity was demonstrated theoretically and empirically with an example of a set of maize crosses spanning a wide range of genetic divergence of parents. Among maize crosses, midparent value predicted 86% of variation in heterosis and inbreeding depression within parent populations predicted 70% of variation in heterosis for grain yield, both lines of evidence supporting a strong linear increase in heterosis with increased heterozygosity due to parental divergence. In the case of inbred lines, the relationship between midparent value and heterosis held suggesting a multi-locus component in which lower yielding inbreds were fixed for recessive alleles at more loci than higher yielding inbred lines. Even though a consistent linear relationship between heterozygosity and heterosis was observed in our empirical test of theory, the exact relationship will vary among species, parents within species, and among phenotypes based on genetic architecture and the level of divergence. The linear relationship between heterosis and heterozygosity is somewhat consistent with Shull’s (1914) original use of the term heterosis as the “stimulating effect of hybridity.” Sprague (1983) suggested that Shull (1914) used the term heterosis to avoid Mendelian connotations, which would have been appropriate given that genetic models of heterosis were undeveloped at the time. However, in the context of modern genetic and quantitative genetic models, one could define heterosis as the “increase in value with an increase in heterozygosity” to capture the continuous relationship with heterozygosity as opposed to a discreet contrast between parental and hybrid generations. One caveat is that heterosis is only linear function of heterozygosity in absence of epistasis. With epistasis, heterosis becomes a nonlinear function of heterozygosity.

Defining heterosis as an increase in value with increased heterozygosity would provide a definition that is the exact opposite of inbreeding depression, which is a reduction in value with a reduction in heterozygosity caused by inbreeding. Heterosis and inbreeding depression are often referred to as opposite effects, although we are not aware of a formal definition of heterosis as a mathematical inverse of inbreeding depression. Mathematically, inbred midparent heterosis was equivalent to inbreeding depression in the F_1_ and F_2_ generations, with values of 2(*p̅*_1_*p̅*_2_ + Δ^2^)*d* and 2p̅_1_p̅_2_Δ^2^*d*, respectively (equations (2) and (3), **Figure 2**) which demonstrates mathematical equivalence of heterosis and inbreeding depression in these two common cross generations. The exact linear relationship between heterosis and inbreeding depression does not hold in general in longer-term evolutionary models in which changes in allele frequency are functions of drift, selection, mutation and migration Charlesworth and Charlesworth (1987); Charlesworth and Charlesworth (2018). 

Whereas inbreeding depression and heterosis are inverse functions of heterozygosity under divergence due to drift, there is one important contrast in context of population structures. In the present model of heterosis the F_2_ generation is taken as a reference population (Falconer and Mackay, 1996; Hill, 1982; Lamkey and Edwards, 1999; Willham and Pollak, 1985), and hence, the inbreeding coefficient, *F*, is assumed to be zero in the F_2_ generation. Heterozygosity in the F_2_ generation is equal to 2*p̅*_1_*p̅*_2_. Heterozygosity in F_1_ generation is 2*p̅*_1_*p̅*_2_ + 2Δ^2^, which exceeds heterozygosity in the F_2_ reference generation by 2Δ^2^. It is appropriate that the F_2_ generation serves as a reference generation because the heterozygosity in the F_2_ generation can be preserved by inter-mating a large number of individuals. Hence, the excess heterozygosity observed in the F_1_ generation represents the transient excess heterozygosity achieved for a single generation and then lost. The excess heterozygosity in the F_1_ generation directly accounts for heterosis that is achieved in the F_1_ generation and lost immediately. Performance of F_1_ hybrids “has been raised to almost mystical status” (Mackay, 2021) because of their unique and valuable performance relative to parents and as evidenced by the attention that heterosis has received in research and breeding. The unique performance of F_1_ hybrids is due in large part to the excess and transient heterozygosity in the F_1_ generation that is lost with additional matings, and with it, the “mystical” performance of the F_1_ hybrid.

A primary objective of our re-interpretation of established models was to reemphasize the separate contributions of population structure and nonadditive gene action to heterosis. Gene action has received immense experimental effort whereas the contribution of population structure has received much less emphasis in past research. One must have population structure that creates contrasts in heterozygosity to obtain a useful hybrid. Modern maize breeding programs, for example, are organized according to heterotic groups which are populations of related inbred lines. Crosses between heterotic groups give rise to hybrids that both mask deleterious effects of inbred parents and give rise to high performing modern hybrids (Tracy and Chandler, 2006). As Tracy and Chandler (2006) point out, the heterotic group population structure was not a pre-existing condition, but was developed over decades of immense breeding effort with a combination of conscious breeding decisions and genetic drift dividing maize germplasm into divergent subpopulations. Development of a successful hybrid breeding system in any species necessarily depends on not only non-additive gene action, but the creation of population structure that maximizes heterozyogisity in the F_1_ generation. The relationship between heterosis and heterozygosity does not depend on magnitude of the dominance coefficient, *d*, relative to the homozygote contrast, *a*, i.e, on the degree of dominance being partial, complete, or overdominant. 

While the F_1_ hybrid in context of the right population structure is a special case in quantitative genetics and breeding, it is not, according to Shull (1908), a special case in nature. Shull (1908) described an open-pollinated field of maize as “a series of complex hybrids”, in which every individual plant was a hybrid, just as are the individuals in modern hybrid cultivars. However, the special case developed in Shull’s work was the ability to reproduce hybrids (noninbred individuals) which are not reproducible in nature. In other words, the ability to permanently regenerate the excess heterozygosity in the F_1_ generation that is transient and unique to the F_1_ generation. Hence, the significance of Shull's (1908) was not heterosis *per se*, but the reproducibility of hybrids through the inbred-hybrid system of breeding. In our modern understanding of genetics, it has become well known that heterozygous genotypes are needed to mask myriad recessive or partially recessive alleles that become exposed during inbreeding (Charlesworth and Charlesworth, 1999; Charlesworth and Willis, 2009), making pure line cultivars in species like maize impractical.

There has been great interest in finding a unifying molecular mechanism to heterosis (Baldauf and Hochholdinger, 2023; Birchler et al., 2003; Blum, 2013; Goff, 2011; Das et al., 2021; Kaeppler, 2011, Kaeppler, 2012; Lippman and Zamir, 2007; Mackay et al., 2021; Rehman et al., 2021; Springer and Stupar, 2007; Swanson-Wagner et al., 2006; Wu et al, 2021). However, the notion of a molecular mechanism of heterosis is incongruent with our interpretation of heterosis as a function of two separate but equally important biological processes, population structure and nonadditive gene action. Billiard et al. (2021) recently argued for a need to integrate population genetics and functional biology in an effort to better understand the molecular and physiological mechanisms of genetic dominance as well as the evolution of dominance. Billiard et al. (2021) reviewed a wide range of molecular and physiological mechanisms of dominance all of which in some way result from nonlinear integration of different levels of the genotype to phenotype map. Their review included a range of mechanisms including allele-specific expression, gene duplication, differential gene expression, nonlinear changes in biochemical flux, and protein interactions. 

Research on molecular and physiological mechanisms of dominance has its roots in the debate between Fisher (1928) and Wright (1929) on the evolution of dominance and has continued to this day. Rather than focusing narrowly on a molecular mechanism of heterosis, which alone does not exist, future research would be better focused on the much broader area of understanding the biological basis for nonadditive gene action, dominance in particular. By training the focus of molecular and or biochemical research on nonadditive gene action as opposed to heterosis, it would be more logically consistent with levels of organization in biology and would potentially enable a wider array of experimental designs. Designing studies of heterosis narrows the scope of experimental designs to those containing contrasts between parental and hybrid generations whereas designing studies of gene action allows for a broader scope of genetic designs to associate molecular features with nonadditive gene effects which potentially includes a very wide array of potential experimental designs. Historically, several mating designs were proposed specifically for the study of gene action, including the North Carolina designs (Comstock and Robinson, 1948, Comstock and Robinson, 1952) and the diallel design (Eberhart and Gardner, 1966; Gardner and Eberhart, 1966; Griffing, 1956) which might not by themselves qualify as heterosis experiments if they do not include parents. However, all are appropriate and valuable designs for study of nonadditive gene action. More recently a much wider array of approaches has been taken to understand the nature of dominance (Billiard et al., 2021). Pursuing a broader focus in basic research will enable such studies to have broader implications and impact and would still generate valuable insights into the gene action contribution to heterosis.

## Data Availability

The raw data supporting the conclusions of this article will be made available by the authors, without undue reservation.
