# Supplementary material for: Dual genetic mechanisms of heterosis: population structure and gene action
Source: Front Plant Sci. 2026 Jan 27;16:1715826. doi: 10.3389/fpls.2025.1715826 (PMC12886449; doi:10.3389/fpls.2025.1715826)
Supplement: Supplementary file 1 [file Table1.docx]

Supplemental Table 1. Generation means for Grain yield (Mg ha^-1^), Ear height (cm), and pant height (cm).

| Pedigree | Generation | Grain Yield | SE | Ear height | SE | Plant Height | SE |
| --- | --- | --- | --- | --- | --- | --- | --- |
| B129 x B114 | F_1_ | 10.60 | 0.26 | 106.72 | 1.72 | 223.24 | 2.43 |
| B129 x B114 | F_2_ | 6.82 | 0.28 | 92.27 | 1.95 | 199.92 | 2.66 |
| B129 x B114 | S_1_ | 6.69 | 0.28 | 89.99 | 1.95 | 197.41 | 2.65 |
| B129 x (B129 x B114) | BC | 7.92 | 0.28 | 94.94 | 1.95 | 203.32 | 2.66 |
| B114 x (B129 x B114) | BC | 6.57 | 0.31 | 90.12 | 2.65 | 202.39 | 3.47 |
| B129 x B116 | F_1_ | 10.48 | 0.28 | 112.15 | 1.95 | 238.86 | 2.66 |
| B129 x B116 | F_2_ | 8.26 | 0.27 | 93.07 | 1.95 | 207.35 | 2.66 |
| B129 x B116 | S_1_ | 6.67 | 0.27 | 94.70 | 1.95 | 205.94 | 2.65 |
| B129 x (B129 x B116) | BC | 7.52 | 0.28 | 100.60 | 1.95 | 214.41 | 2.71 |
| B116 x (B129 x B116) | BC | 7.79 | 0.28 | 101.75 | 1.95 | 230.65 | 2.66 |
| B73 x Mo17 | F_1_ | 9.30 | 0.25 | 118.36 | 1.64 | 233.97 | 2.31 |
| B73 x Mo17 | F_2_ | 5.99 | 0.27 | 99.88 | 1.95 | 209.39 | 2.65 |
| B73 x Mo17 | S_1_ | 6.17 | 0.24 | 102.11 | 1.59 | 211.97 | 2.25 |
| B73 x (B73 x Mo17) | BC | 7.22 | 0.28 | 107.56 | 1.95 | 214.80 | 2.66 |
| Mo17 x (B73 x Mo17) | BC | 6.16 | 0.28 | 102.05 | 2.06 | 209.69 | 2.72 |
| SGI912 x BX010 | F_1_ | 11.01 | 0.29 | 118.08 | 2.12 | 246.24 | 2.88 |
| SGI912 x BX010 | F_2_ | 6.94 | 0.27 | 102.56 | 1.95 | 219.06 | 2.65 |
| SGI912 x BX010 | S_1_ | 7.07 | 0.27 | 104.13 | 1.95 | 217.51 | 2.65 |
| SGI912 x (SGI912 x BX010) | BC | 8.47 | 0.28 | 101.59 | 2.06 | 214.42 | 2.82 |
| BX010 x (SGI912 x BX010) | BC | 6.33 | 0.28 | 102.49 | 2.06 | 221.24 | 2.77 |
| SGI912 x B116 | F_1_ | 11.13 | 0.25 | 111.68 | 1.78 | 250.74 | 2.48 |
| SGI912 x B116 | F_2_ | 7.67 | 0.28 | 101.64 | 1.95 | 221.28 | 2.65 |
| SGI912 x B116 | S_1_ | 7.50 | 0.28 | 100.33 | 1.95 | 217.44 | 2.65 |
| SGI912 x (SGI912 x B116) | BC | 8.11 | 0.28 | 101.24 | 1.95 | 214.12 | 2.66 |
| B116 x (SGI912 x B116) | BC | 6.68 | 0.28 | 102.74 | 2.05 | 231.03 | 2.77 |
| TR7245 x B116 | F_1_ | 10.59 | 0.24 | 114.87 | 1.60 | 242.97 | 2.28 |
| TR7245 x B116 | F_2_ | 7.25 | 0.28 | 103.04 | 1.95 | 217.15 | 2.65 |
| TR7245 x B116 | S_1_ | 7.30 | 0.24 | 101.75 | 1.59 | 219.93 | 2.26 |
| TR7245 x (TR7245 x B116) | BC | 8.61 | 0.24 | 109.43 | 1.60 | 218.02 | 2.26 |
| B116 x (TR7245 x B116) | BC | 7.13 | 0.28 | 98.73 | 1.95 | 226.81 | 2.65 |
| BS11(FR)C15 x B129 | F_1_ | 10.12 | 0.28 | 112.54 | 1.95 | 228.65 | 2.67 |
| BS11(FR)C15 x B129 | F_2_ | 7.68 | 0.27 | 105.51 | 2.00 | 215.13 | 2.65 |
| BS11(FR)C15 x B129 | S_1_ | 5.99 | 0.28 | 103.35 | 2.00 | 208.24 | 2.65 |
| BS11(FR)C15 x (BS11(FR)C15 x B129) | BC | 8.00 | 0.28 | 108.57 | 2.00 | 223.97 | 2.76 |
| B129 x (BS11(FR)C15 x B129) | BC | 7.32 | 0.27 | 108.65 | 1.95 | 215.39 | 2.66 |
| BSCB1(R)C16 x B129 | F_1_ | 9.74 | 0.28 | 103.01 | 1.95 | 219.81 | 2.67 |
| BSCB1(R)C16 x B129 | F_2_ | 7.31 | 0.28 | 96.81 | 1.95 | 208.48 | 2.65 |
| BSCB1(R)C16 x B129 | S_1_ | 6.45 | 0.28 | 91.85 | 1.95 | 206.10 | 2.77 |
| BSCB1(R)C16 x (BSCB1(R)C16 x B129) | BC | 7.66 | 0.28 | 94.64 | 1.95 | 208.71 | 2.66 |
| B129 x (BSCB1(R)C16 x B129) | BC | 7.32 | 0.27 | 99.41 | 1.95 | 209.66 | 2.66 |
| BSKRL4(HI)C2 x B129 | F_1_ | 9.93 | 0.27 | 111.42 | 1.95 | 230.06 | 2.67 |
| BSKRL4(HI)C2 x B129 | F_2_ | 7.08 | 0.28 | 104.22 | 1.95 | 216.55 | 2.65 |
| BSKRL4(HI)C2 x B129 | S_1_ | 6.39 | 0.27 | 98.76 | 1.95 | 212.63 | 2.65 |
| BSKRL4(HI)C2 x (BSKRL4(HI)C2 x B129) | BC | 7.00 | 0.27 | 101.12 | 1.95 | 219.67 | 2.66 |
| B129 x (BSKRL4(HI)C2 x B129) | BC | 7.63 | 0.28 | 103.30 | 1.95 | 216.02 | 2.66 |
| BS13(S)C10 x BSCB1(R)C15 | F_1_ | 9.55 | 0.28 | 99.67 | 2.00 | 220.10 | 2.67 |
| BS13(S)C10 x BSCB1(R)C15 | F_2_ | 7.36 | 0.28 | 92.09 | 1.95 | 209.15 | 2.70 |
| BS13(S)C10 x BSCB1(R)C15 | S_1_ | 6.27 | 0.28 | 89.45 | 2.08 | 199.26 | 2.65 |
| BS13(S)C10 x (BS13(S)C10 x BSCB1(R)C15) | BC | 8.01 | 0.28 | 92.70 | 1.95 | 202.37 | 2.66 |
| BSCB1(R)C15 x (BS13(S)C10 x BSCB1(R)C15) | BC | 7.45 | 0.28 | 93.58 | 1.95 | 211.44 | 2.71 |
| BS13(S)C10 x BSSS(R)C15 | F_1_ | 9.05 | 0.28 | 106.07 | 1.95 | 220.61 | 2.67 |
| BS13(S)C10 x BSSS(R)C15 | F_2_ | 7.81 | 0.28 | 98.83 | 2.00 | 209.78 | 2.72 |
| BS13(S)C10 x BSSS(R)C15 | S_1_ | 6.70 | 0.28 | 92.08 | 1.95 | 198.28 | 2.65 |
| BS13(S)C10 x (BS13(S)C10 x BSSS(R)C15) | BC | 8.22 | 0.28 | 97.45 | 1.95 | 205.52 | 2.66 |
| BSSS(R)C15 x (BS13(S)C10 x BSSS(R)C15) | BC | 7.71 | 0.27 | 98.36 | 1.95 | 215.31 | 2.66 |
| BSSS(R)C15 x BSCB1(R)C15 | F_1_ | 8.88 | 0.28 | 99.93 | 1.95 | 224.36 | 2.67 |
| BSSS(R)C15 x BSCB1(R)C15 | F_2_ | 7.44 | 0.27 | 98.95 | 1.95 | 216.06 | 2.65 |
| BSSS(R)C15 x BSCB1(R)C15 | S_1_ | 5.72 | 0.28 | 95.98 | 1.95 | 209.60 | 2.65 |
| BSSS(R)C15 x (BSSS(R)C15 x BSCB1(R)C15) | BC | 7.74 | 0.28 | 108.05 | 1.95 | 230.62 | 2.66 |
| BSCB1(R)C15 x (BSSS(R)C15 x BSCB1(R)C15) | BC | 6.80 | 0.28 | 94.03 | 1.95 | 212.08 | 2.66 |
| B114 | Parent | 2.70 | 0.35 | 66.37 | 1.99 | 157.87 | 2.72 |
| B116 | Parent | 3.66 | 0.24 | 85.15 | 1.47 | 209.33 | 2.14 |
| B129 | Parent | 4.36 | 0.23 | 86.51 | 1.36 | 182.21 | 2.02 |
| B73 | Parent | 4.99 | 0.29 | 96.51 | 1.95 | 190.16 | 2.67 |
| Mo17 | Parent | 4.30 | 0.29 | 84.36 | 1.95 | 180.13 | 2.67 |
| BX010 | Parent | 4.02 | 0.33 | 90.74 | 1.95 | 192.53 | 2.67 |
| SGI912 | Parent | 6.05 | 0.25 | 83.20 | 1.60 | 175.44 | 2.29 |
| TR7245 | Parent | 6.15 | 0.28 | 94.47 | 1.95 | 182.99 | 2.67 |
| BS11(FR)C15 | Parent | 6.66 | 0.28 | 109.88 | 2.01 | 227.89 | 2.68 |
| BS11(FR)C15 | S_1_ | 4.69 | 0.28 | 97.17 | 1.95 | 204.12 | 2.65 |
| BSCB1(R)C16 | Parent | 6.03 | 0.29 | 81.60 | 1.96 | 188.87 | 2.69 |
| BSCB1(R)C16 | S_1_ | 4.01 | 0.28 | 72.63 | 1.95 | 170.04 | 2.65 |
| BSKRL4(HI)C2 | Parent | 4.59 | 0.26 | 92.07 | 1.62 | 205.97 | 2.33 |
| BSKRL4(HI)C2 | S_1_ | 3.36 | 0.30 | 83.97 | 1.95 | 192.32 | 2.65 |
| BS13(S)C10 | Parent | 7.94 | 0.25 | 87.63 | 1.61 | 183.36 | 2.33 |
| BS13(S)C10 | S_1_ | 6.95 | 0.24 | 83.36 | 1.61 | 175.46 | 2.28 |
| BSCB1(R)C15 | Parent | 5.64 | 0.25 | 82.07 | 1.63 | 192.33 | 2.34 |
| BSCB1(R)C15 | S_1_ | 4.17 | 0.25 | 74.75 | 1.59 | 176.01 | 2.26 |
| BSSS(R)C15 | Parent | 6.67 | 0.25 | 99.79 | 1.62 | 216.84 | 2.33 |
| BSSS(R)C15 | S_1_ | 4.94 | 0.25 | 88.93 | 1.59 | 200.03 | 2.26 |
